# Resource Utilization and Cost Analysis of Pediatric Esophageal Foreign Bodies

**DOI:** 10.1177/19160216251318256

**Published:** 2025-02-10

**Authors:** Tanya Chen, Jennifer M. Siu, Yasmine Madan, Gar-Way Ma, Peter J. Gill, Nicholas Carman, Evan J. Propst, Nikolaus E. Wolter

**Affiliations:** 1 Department of Otolaryngology—Head and Neck Surgery, Hospital for Sick Children, University of Toronto, Toronto, ON, Canada; 2 Department of Paediatrics, Hospital for Sick Children, University of Toronto, Toronto, ON, Canada; 3 Department of Gastroenterology, Hepatology and Nutrition, Hospital for Sick Children, University of Toronto, Toronto, ON, Canada

**Keywords:** pediatric otolaryngology, bronchoesophagology, cost effectiveness, systematic review, esophagology, laryngology

## Abstract

**Objective:**

Impacted esophageal foreign bodies (EFBs) are a common but preventable presentation in children, requiring prompt removal in the operating room by esophagoscopy. Our objective was to describe the overall cost of impacted pediatric EFBs and determine factors that increase resource burden.

**Methods:**

A cost analysis of pediatric patients undergoing esophagoscopy for EFB removal from 2010 to 2021 was performed. Characteristics of each EFB, patient transfer, and hospital course were collected. Direct and indirect healthcare costs were calculated using hospital-specific costs and provincial fees. Amounts were calculated in Canadian dollars.

**Results:**

Six hundred and eighty patients were included. The total amount spent on pediatric EFBs from 2010 to 2021 was $2,673,288. The mean total cost per child with an EFB was $3469. An extra hour of delay before Otolaryngology—Head and Neck Surgery (OHNS) consultation at a tertiary hospital corresponded to an $816 cost [95% confidence interval (CI; 244.7–1287.4)]. On average, children requiring transfer to a tertiary care center cost $1965 more than those initially presenting to a tertiary care center (*P* = .001). Higher-risk EFBs (n = 165, 24%) were associated with a longer hospital stay and greater complication rate and resulted in a $4095 increase in overall cost compared to lower-risk EFBs [$6829 (standard deviation (SD) $11,347) vs $2734 (SD $10,451), *P* = .02]. Button battery ingestions cost 8.8 times more than non-dangerous EFBs, such as coins. Longer distance for transfer was associated with a higher likelihood of having complications [odds ratios (OR) 1.5, 95% CI (1.1–1.8)].

**Conclusion:**

EFBs pose a significant economic burden to the healthcare system, driven by transfer to a tertiary care center, delays in transfer to the operating room, and high-risk EFBs. It is critical to identify areas for improved efficiency such as increased parental education for primary prevention, early involvement of the OHNS team and improving the capacity of community hospitals to manage EFB to limit transfers when possible.

## Graphical abstract

**Graphical Abstract:**
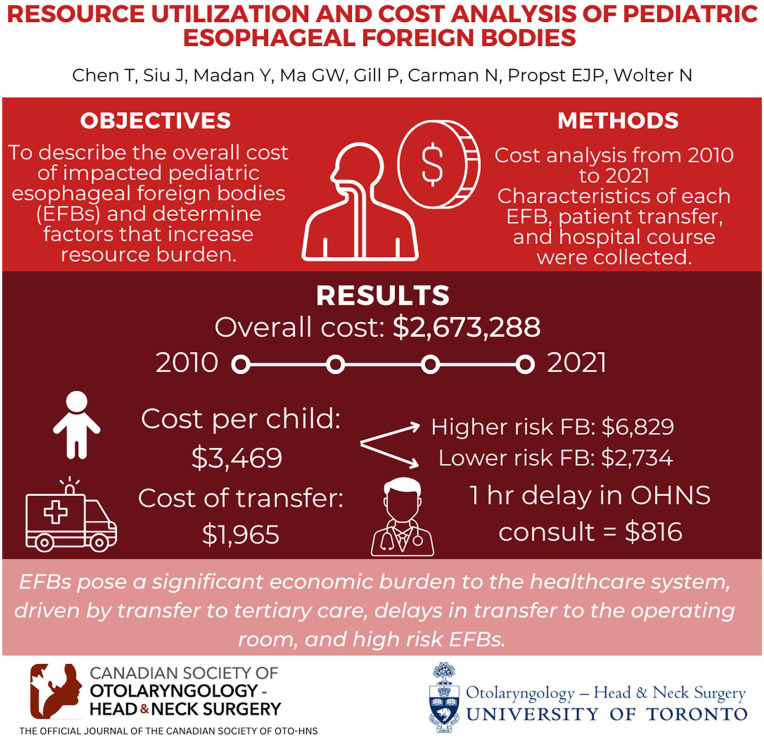

## Key Message

• Delays in care result in higher rates of complications and higher costs for pediatric esophagoscopy.
• Transfers to specialized pediatric centers are not always necessary and result in prolonged impaction time.
• Targeted referral pathways and protocols are necessary to optimize care and reduce costs.

## Background

Esophageal foreign body (EFB) impaction represents a common, preventable, but potentially serious issue in children. Over 100,000 EFB impactions occur worldwide per year and 75% occur in children below the age of six years.^1–3^ The incidence of pediatric EFB ingestion has been increasing in recent years, with higher rates of dangerous EFBs such as button batteries.^4,5^ After EFB ingestion, prompt management is crucial to prevent complications, such as esophageal perforation and damage to adjacent structures, which can result in a prolonged hospital stay and further interventions.^6,7^ The majority of impacted EFBs are removed endoscopically via rigid or flexible esophagoscopy. While most pediatric EFBs are uncomplicated, many children are transferred to tertiary pediatric centers due to specialized equipment needs and surgeon comfort.^8^ In the province of Ontario, there are only four tertiary pediatric centers that care for nearly 1.9 million children under the age of 12.^9^ In our region, the majority of all pediatric EFBs are transferred to our center and managed by the Otolaryngology—Head and Neck Surgery (OHNS) service. Due to this large catchment area, there are often delays in care due to transfer, which can create frustration for patients and their families, as well as incur significant costs for both the family and the healthcare system.

Cost considerations for pediatric EFBs include the direct procedural costs of esophagoscopy and expenses such as interhospital transfer, operating room resources, involvement of consultation services, nursing care staffing, management and supportive services, and housekeeping. These expenses increase dramatically in the event of complications from the EFB. Increasing constraints on healthcare resources demand a close examination of the care pathway of these children and an analysis of the economic dimensions of esophagoscopy for EFB removal in this context.

To date, there are no studies characterizing the care pathways and costs associated with patients with impacted EFB and the various factors that influence their treatment and use of resources. The objectives of this study were to describe the costs associated with pediatric EFB and identify potential areas to reduce unnecessary expenses. By analyzing specific time points in the care of these patients and highlighting the most resource-intensive time points, targetable areas of improvement could potentially be identified which may reduce the morbidity of EFB and reduce the financial burden on the healthcare system.

## Methods

Following research ethics approval (REB# 1000079943), a retrospective chart review identified 680 pediatric patients undergoing esophagoscopy for EFB removal at a tertiary care pediatric center from January 1, 2010 to December 31, 2021. Patients were included if they were less than 18 years of age at the time of presentation and underwent rigid esophagoscopy for possible EFB removal by OHNS. All patients with EFB are managed by the OHNS service in our center and the pediatric gastroenterology service is involved on an as-needed basis. Patients who underwent esophagoscopy where an EFB was not identified were included in statistical analyses given the perioperative risks associated with esophagoscopy alone. Exclusion criteria were children undergoing elective esophagoscopy for biopsy, dilation, hemostasis, or any intervention that did not include EFB removal. If there was a concurrent biopsy or other procedure at the time of EFB removal, these patients were still included.

Information collected included sex, age, race, type of EFB, method of transport to the initial hospital, method of transport to tertiary care hospital, imaging on arrival, consulting services, admission location, perioperative details, length of stay, and complications. Appendix 1 depicts the geographical area of referral centers in relation to our tertiary care pediatric center in the province of Ontario. The degree of EFB risk was derived from the 2015 North American Society For Pediatric Gastroenterology, Hepatology & Nutrition position paper on pediatric foreign body ingestion,^10^ with higher-risk EFB defined as magnets (2 or more), batteries, sharp objects, or bones,^8^ and all other EFB considered lower risk, including coins, blunt objects, food, or no identified EFB. A complicated course was defined as any of the following: admission or transfer to the intensive care unit (ICU), length of stay > 48 hours, intraoperative complications such as hemodynamic instability or airway management challenges, or postoperative complications such as hemodynamic instability, postoperative radiological abnormalities, return to hospital within 1 week of discharge, or death. All patient charts and the entire inpatient hospital course were reviewed thoroughly, including daily progress notes and nursing notes.

Multiple index time periods in the EFB care pathway were measured: ingestion to triage at community hospital, triage to community hospital Emergency Department (ED) assessment, community hospital ED assessment to tertiary care hospital contact, tertiary care hospital contact to arrival at tertiary care hospital ED, tertiary care hospital ED arrival to repeat imaging, tertiary care hospital ED arrival to OHNS consult, tertiary care hospital ED arrival to operating room, length of surgery, and overall admission time. The length of surgery was calculated between the start of anesthesia and the time of extubation. Data was extracted for each patient via electronic medical record and paper chart review.

### Cost Data

The cost of each EFB was calculated by summing all expenses associated with each of the index time periods in the EFB care pathway. Both direct and indirect costs were calculated for each step with information obtained through the Enterprise and Data Analytics Department at our hospital. Direct costs were those incurred by the patient, such as imaging, consultations, bloodwork, and operating room expenses. Indirect costs were non-patient–related expenses, such as management and supportive services, housekeeping, etc. Mean cost estimates were provided by the Business Department at the Hospital for Sick Children. Transportation costs for land ambulances were obtained from paramedic services of each region in Ontario (Durham, Peel, York, Waterloo, Halton, and Niagara), as well as 15 different municipalities for each community hospital that was represented in the dataset. Costs for air ambulance transportation were obtained by contacting the Ornge Statistics Centre for hospital-specific data during the inclusion period of this study.

### Statistical Analyses

Descriptive characteristics were performed for demographic variables and associated costs with location and type of admission. Independent *t*-tests and subgroup analyses were used to compare patients presenting to a community hospital versus patients presenting to a tertiary care hospital, as well as higher-risk versus lower-risk EFBs. Both linear and logistic regression were performed to ascertain the effect of continuous variables (various time delays in care pathway, distance of transfer) on the likelihood of complication and overall cost using odds ratios (OR) and 95% CIs. Other covariates included age and sex. Analysis was performed using SPSS version 26.0 (SPSS, Chicago, IL).

## Results

### General Patient Population Characteristics

**Figure 1.**
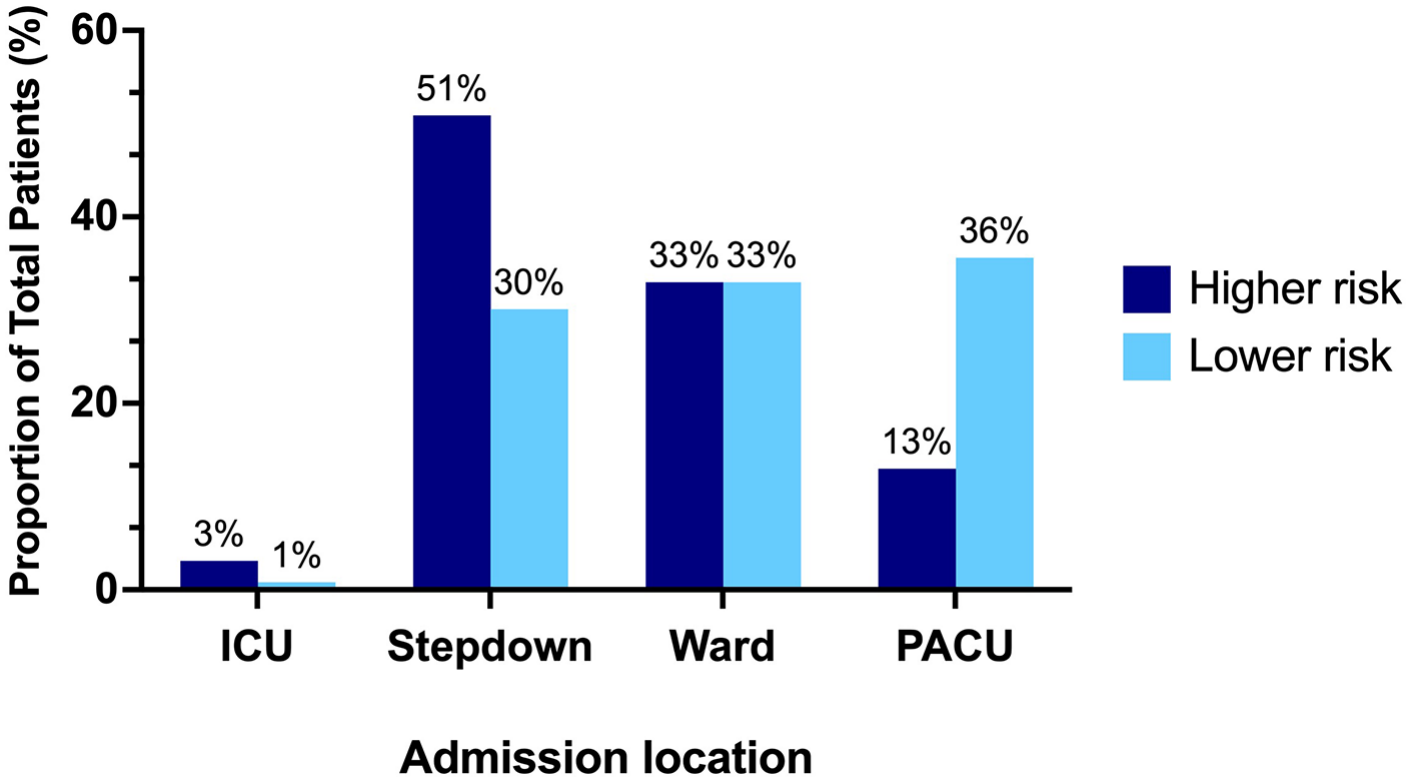
Locations of admission according to foreign body type.

Most patients (77.9%, 530/680) presented initially to a community hospital and were transferred to a tertiary care hospital for EFB removal (Table 1). Of the patients who presented to a community hospital, 44.3% (235/530) were transported via ambulance to the tertiary care hospital (233 by land, two by air). Of those where the mode of transportation was recorded, 83.8% (326/389) were driven from the community hospital to the tertiary care hospital by their family. Higher-risk EFBs were seen in 24.3% (165/680) of patients. Consultation from at least one other service in addition to OHNS was required in 23.7% (161/680) of patients, the most common being gastroenterology. X-ray was performed at the community hospital in 90% (612/680) of patients, with 70.2% (372/530) of patients having repeat imaging upon arrival to the tertiary care hospital. Over 50% (87/165) of higher-risk EFBs required postoperative admission to a higher monitored care setting such as a stepdown unit or ICU (Figure 1). Lower-risk EFB ingestions were admitted in equal numbers to a stepdown unit, the regular ward, or discharged from the PACU. When comparing lower-risk EFBs that were discharged from PACU versus ward admission, the major notable difference was the time at the end of surgery. 87.9% (160/182) of lower-risk PACU discharges had surgery completed between 8 AM and 8 PM, whereas 55.2% (90/163) of those admitted to the ward had surgery completed overnight (8 PM to 8 AM). Children who were admitted to the ward were also younger than those who were discharged from PACU [mean (standard deviation, SD) = 4.9 (1.2) vs 4.1 (1.6) years old]. Only 13.3% (22/165) of higher-risk EFBs were discharged directly from the PACU compared to 35.5% of lower-risk EFBs (183/515).

**Table 1.** Patient Demographic and Admission Details.

	Total	Presenting to community hospital			Presenting to tertiary hospital		
		All	Higher risk	Lower risk	All	Higher risk	Lower risk
All esophageal foreign bodies	680	530 (77.9)	120 (22.6)	410 (77.4)	150 (22.1)	45 (30.0)	105 (70.0)
Method of transport to initial hospital, n (%)							
Land ambulance	73 (10.7)	63 (11.9)	9 (7.5)	54 (13.2)	10 (6.6)	2 (4.4)	8 (7.6)
Parent transport	426 (62.7)	326 (61.5)	80 (66.7)	246 (60.0)	100 (66.7)	32 (71.2)	68 (64.8)
Unknown	181 (26.6)	141 (26.6)	31 (25.8)	110 (26.8)	40 (26.7)	11 (24.4)	29 (27.6)
Method of transport to tertiary care, n (%)							
Land ambulance	233 (44.0)	233 (44.0)	61 (50.8)	172 (42.0)	0	0	0
Air ambulance	2 (0.3)	2 (0.3)	2 (1.7)	0 (0.0)	0	0	0
Parent transport	127 (24.0)	127 (24.0)	33 (27.5)	94 (22.9)	0	0	0
Unknown	168 (31.7)	168 (31.7)	96 (5.0)	144 (35.1)	0	0	0
Imaging on arrival to initial ER, n(%)							
X-ray	612 (90.0)	484 (91.3)	96 (80.0)	388 (94.6)	128 (85.3)	37 (82.2)	91 (86.7)
CT	5 (0.9)	5 (1.0)	3 (2.5)	2 (0.5)	0	0	0
No imaging	63 (9.3)	41 (7.7)	21 (17.5)	20 (4.9)	22 (14.7)	8 (17.8)	14 (13.3)
Consulting services, n(%)							
Gastroenterology	64 (9.4)	42 (7.9)	25 (20.8)	17 (4.1)	22 (14.7)	12 (26.7)	10 (9.5)
General surgery	38 (5.6)	15 (2.8)	11 (9.2)	4 (1.0)	23 (15.3)	14 (31.1)	9 (8.6)
Pediatric medicine	16 (2.4)	12 (2.3)	8 (6.7)	4 (1.0)	4 (2.7)	1 (2.2)	3 (2.9)
Psychiatry	4 (0.6)	4 (0.7)	3 (2.5)	1 (0.2)	0	0	0
Other	39 (5.7)	32 (6.0)	20 (16.7)	12 (2.9)	7 (4.7)	2 (4.4)	5 (4.8)
Admission location, n (%)							
ICU	11 (1.6)	10 (1.9)	5 (4.2)	5 (1.2)	1 (0.7)	0 (0.0)	1 (1.0)
Stepdown unit	233 (34.3)	179 (33.8)	65 (54.2)	114 (27.8)	54 (36.0)	17 (37.8)	37 (35.2)
Ward	216 (31.8)	163 (30.7)	35 (29.2)	124 (30.2)	53 (35.3)	18 (40.0)	39 (37.1)
Discharge from PACU	204 (30.0)	163 (30.7)	13 (10.8)	150 (36.6)	41 (27.3)	9 (20.0)	32 (30.5)
Unknown	16 (2.3)	15 (2.8)	1 (0.8)	14 (3.4)	1 (0.7)	1 (2.2)	0
Mean (SD) length of stay by location (h)							
ICU	395.1 (361.2)	432.4 (357.6)	454.4 (355.9)	410.4 (399.9)	21.8	0	21.8
Stepdown unit	28.5 (42.7)	30.2 (46.4)	61.6 (65.5)	15.7 (22.9)	22.3 (24.2)	31.4 (28.4)	18.7 (21.8)
Ward	19.8 (22.4)	19.0 (24.1)	40.6 (41.3)	12.7 (9.4)	21.9 (16.9)	28.5 (19.1)	18.7 (14.8)
Discharge from PACU	6.4 (10.0)	6.6 (10.8)	6.1 (10.2)	6.7 (10.9)	5.8 (7.9)	8.7 (9.7)	5.0 (7.3)
Mean (SD) time presentation to OR (h)	13.5 (12.4)	13.7 (13.0)	11.2 (14.3)	14.7 (8.1)	13.1 (8.3)	13.3 (9.1)	12.3 (7.8)
Complications, n (%)							
Present	246 (36.2)	187 (76.0)	77 (41.2)	110 (58.8)	59 (24.0)	24 (40.7)	35 (59.3)
ICU	11 (4.5)	10 (5.3)	5 (6.5)	5 (4.5)	1 (1.7)	0	1 (2.8)
LOS > 48 h	57 (23.2)	46 (24.6)	30 (38.9)	16 (14.5)	11 (18.6)	6 (25.0)	5 (14.3)
Intraoperative complications	97 (39.4)	59 (31.6)	40 (51.9)	19 (17.3)	38 (64.4)	20 (83.3)	18 (51.4)
Postoperative complications	81(32.9)	49 (26.2)	31 (40.3)	18 (16.4)	32 (54.2)	19 (79.2)	13 (37.1)
Not present	434 (63.8)	335 (77.2)	45 (13.4)	290 (86.6)	99 (22.8)	22 (22.2)	77 (77.8)

Abbreviations: CT, computed tomography; EFB, esophageal foreign body; ICU, intensive care unit; LOS, length of stay; MRI, magnetic resonance imaging; OR, operating room; PACU, postoperative care unit; SD, standard deviation.

### Timeline of Care

**Figure 2.**
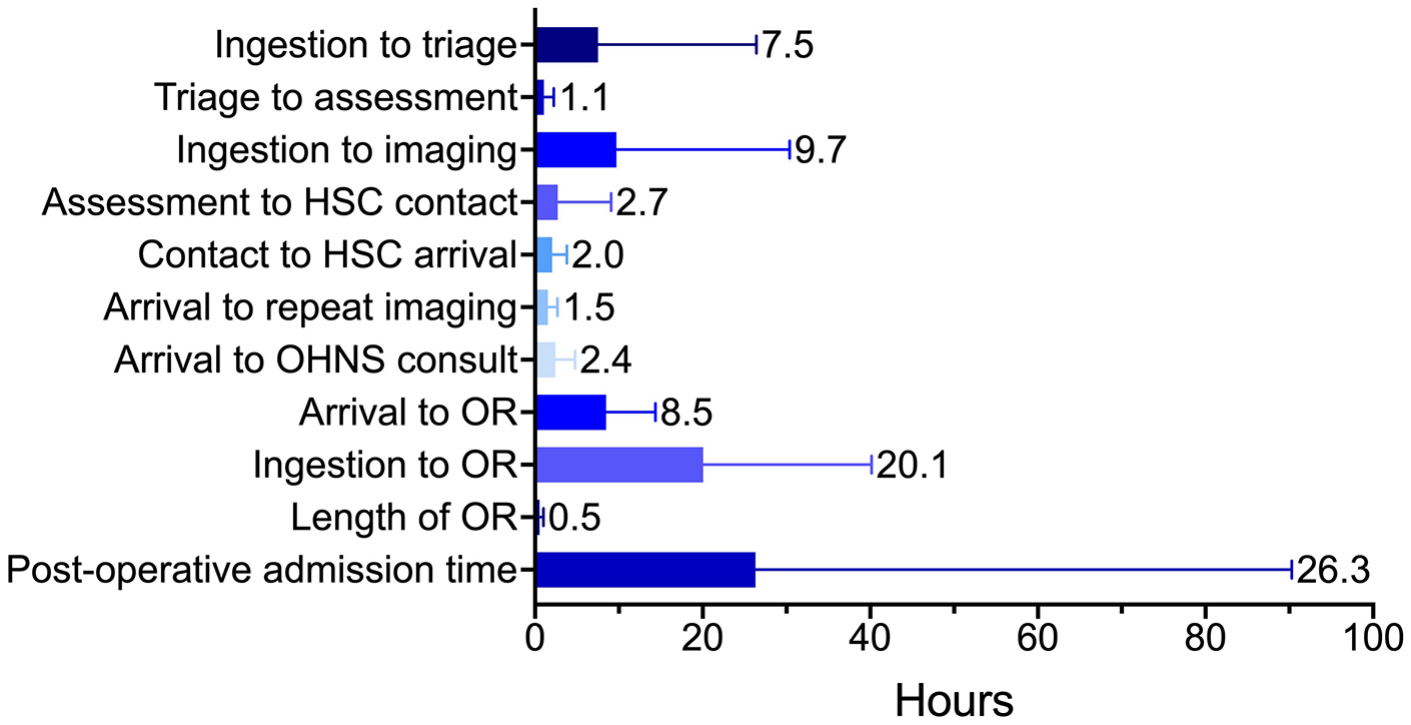
Mean time elapsed between various points in timeline of care of pediatric esophageal foreign bodies. Abbreviations: ED, emergency department; OHNS, otolaryngology—head and neck surgery; OR, operating room.

The mean (SD) overall time from initial presentation to removal was 14.1 (12.4) hours. The mean (SD) time from presentation at a community hospital to removal at a tertiary care hospital was 8.5 (6.0) hours and the mean time from presentation at a tertiary care hospital to removal at the same institution was 4.6 (3.3) hours (Table 1). The mean (SD) time from ingestion to presentation to the ED was 7.5 (18.9) hours. However, only 58.1% (395/680) of parents reported an approximate time of ingestion because the majority were unwitnessed. Figure 2 characterizes times between index timepoints for EFBs. There was a mean (SD) time of 2.4 (2.3) hours from patient arrival to the tertiary hospital ED and contact with the OHNS team. For patients presenting to community hospitals, the OHNS team at the tertiary care hospital was contacted preemptively prior to transfer in only 47.9% (254/530) of transferred patients. Where OHNS was called prior to transfer, the mean (SD) time to consultation upon arrival in the tertiary care ED was 1.9 (1.8) hours, compared to 3.1 (2.7) hours when OHNS was not called prior to transfer.

The median transfer distance was 40.4 km (interquartile range: 32.7), ranging from 0.3 to 787.0 km. This corresponded to an average delay of 5.0 (6.1) hours from arrival at community hospital to arrival at tertiary care hospital. Two hundred and thirty-five children (44.3%) presenting to a community hospital were transferred via land or air ambulance. For higher-risk EFBs, the mean (SD) delay was 4.4 hours (2.6), compared to 6.8 hours (11.7) for lower-risk EFBs. Longer distance for transfer was associated with a higher likelihood of having complications [OR 1.5, 95% confidence interval (CI; 1.1–1.8)]. This was similarly true for duration of transfer from the community hospital to arrival at the tertiary hospital ED [OR 1.3, 95% CI (1.0–1.7)]. Repeat imaging after arrival to tertiary care was performed in 70.2% (372/530) of patients, resulting in an average increase in time to removal of 2.4 hours. The mean (SD) duration of rigid esophagoscopy for EFB removal was 32.1 (28.3) minutes, ranging from 9 minutes to 5.3 hours. Seventy percent (476/680) of patients were admitted to hospital post-operatively, and 30.0% (204/680) were discharged from the postoperative care unit (PACU; Table 1). The mean (SD) length of stay (LOS) for lower-risk EFB patients was 18.2 (59.1) hours compared to 48.9 (71.4) hours for higher-risk EFB (*P* = .02).

### Costs Associated With EFB Management

The total cost of EFB management in the Greater Toronto Area during the study period was $2,673,288, including interhospital transportation fees of $34,404. The mean (SD) cost per year was $222,774 ($113,732). The mean (SD) total cost per child with EFB was $3469 (589). The mean (SD) total cost per patient transferred to the tertiary care hospital for treatment was $4265 (12,340) compared to $2300 (2430) for those initially presenting to the tertiary care hospital (Table 2). Delay from arrival at the tertiary care hospital to OHNS consult was a statistically significant predictor of cost [*R*^2^ = 19.7, *F*(1613) = 7.9, *P* = .005]. Each additional hour of delay prior to OHNS consultation corresponded to an $816 increase in cost [95% CI (244.7–1287.4)]. Each additional hour of delay from arrival to the tertiary care hospital to OR start corresponded to a $133 increase in cost [95% CI (78.1–299.8)], but this was not statistically significant (*P* = .086). Length of surgery predicted 13.0% of the variation in cost [*F*(1614) = 68.4, *P* = .005])]. Each additional minute of OR time led to a $119 increase in overall cost [95% CI (91.0–146.9)].

**Table 2.** Cost of EFB Based on Type of Foreign Body and Admission Details.

Presentation	n	Presenting to community hospital Cost [mean (SD), $]	n	Presenting to tertiary hospital Cost [mean (SD), $]	*P*-value
Total	453	4265.88 (12,340.95)	146	2300.04 (2430.47)	.001
Higher-risk EFB	111	8249.19 (12,987.68)	43	3162.18 (2870.50)	<.001
Lower-risk EFB	342	2973.05 (11,858.34)	103	1940.12 (2135.52)	.04
Post-op admission to ICU					
Total	7	81,147.07 (54,503.66)	1	4400.09 (N/A)	N/A
Higher-risk EFB	3	64,897.08 (38,980.81)	0	N/A	
Lower-risk EFB	4	93,334.56 (66,829.53)	1	4400.09 (N/A)	
Post-op admission to stepdown					
Total	172	4740.04 (6215.86)	55	2776.20 (2372.36)	<.001
Higher-risk EFB	64	8346.80 (8142.83)	18	4043.45 (3460.23)	
Lower-risk EFB	108	2602.69 (3211.73)	37	2159.71 (1262.74)	
Post-op admission to ward					
Total	157	2725.77 (2912.27)	57	2684.42 (2720.96)	.649
Higher-risk EFB	36	5018.88 (5028.29)	17	3423.99 (2156.34)	
Lower-risk EFB	121	2043.52 (1266.68)	40	2370.10 (2895.42)	
Post-op discharge from PACU					
Total	117	1035.74 (822.08)	33	778.88 (1065.98)	.335
Higher-risk EFB	8	761.64 (516.39)	8	623.00 (448.01)	
Lower-risk EFB	109	1055.86 (838.22)	25	828.76 (1202.44)	

Abbreviations: EFB, esophageal foreign body; ICU, intensive care unit; PACU, postoperative care unit; SD, standard deviation.

**Figure 3.**
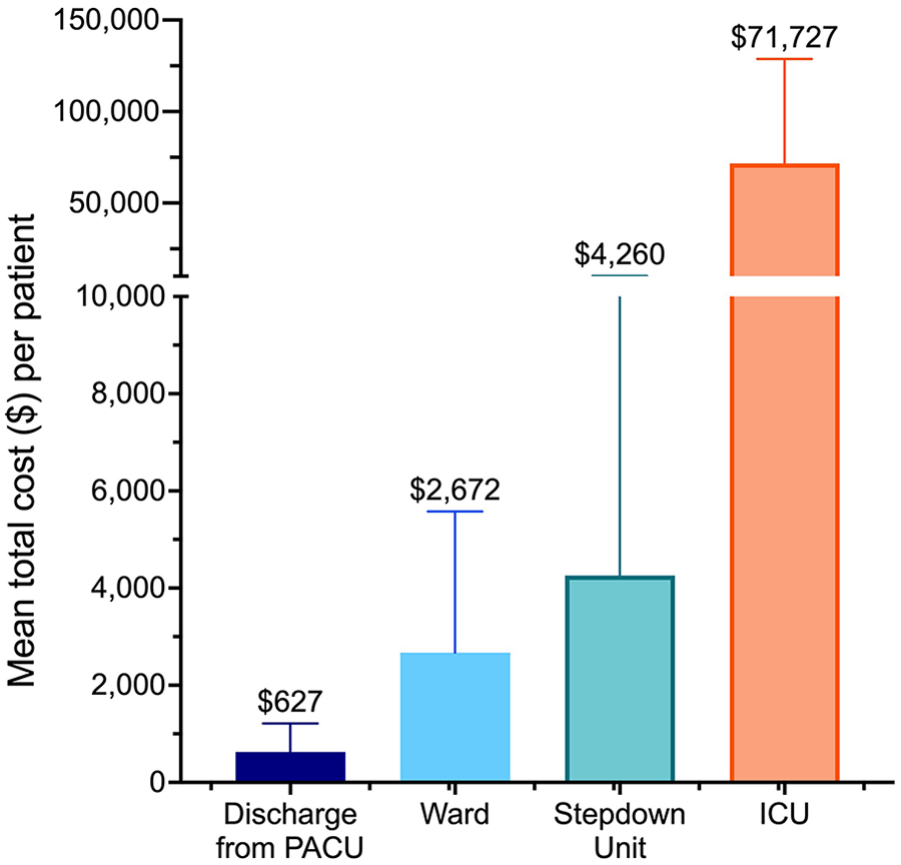
Mean total cost per patient varied by location of admission of pediatric EFB patients. Abbreviations: ICU, intensive care unit; PACU, postoperative care unit.

The mean cost per day for admission was $7225 to the ICU, $3016 to the stepdown unit, $2756 to the ward, and $1064 for discharge directly from the PACU. Five of the 11 ICU admissions were due to a button battery impaction. Figure 3 demonstrates the average cost burden according to location of hospital admission. Thirty-six percent (246/680) of all patients had a complicated course. Patients with complications had a mean (SD) LOS of 2.2 (0.4) days, corresponding to a mean (SD) cost of $5868 (1519) per patient, whereas those without complications had a mean (SD) LOS of 13.8 (6.9) hours, corresponding to a mean (SD) cost of $1483 (289) per patient. Table 2 describes the cost differences according to initial hospital presentation and type of FB ingested. Of all patients who presented initially to a community hospital, 75.5% (342/453) were lower-risk EFBs and were on average $1965 more costly per patient than the lower-risk EFBs that presented initially to a tertiary hospital (Table 2). Though hospital-specific cost data was not available for the community hospitals, assuming these lower-risk EFBs could be removed at their initial presenting community hospital, this would have resulted in a minimum cost savings of $246,111 over the study period which represents 9.2% of the total cost spending. This only accounted for additional ED assessments and transfer costs, but does not include the costs for operational costs at the initial hospital.

**Figure 4.**
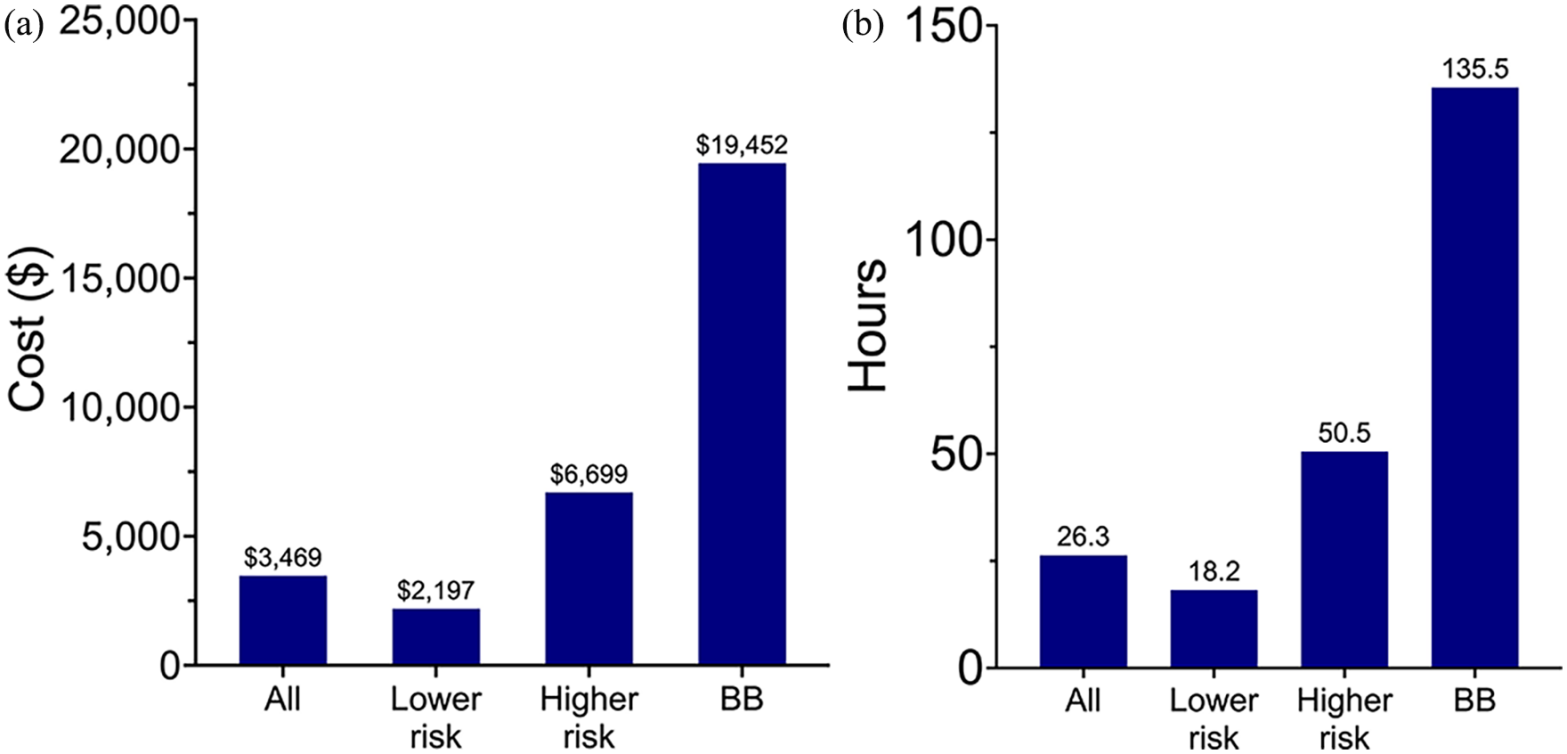
Cost and duration of stay vary between higher and lower-risk EFB. (A) Cost according to type of EFB. (B) Duration of stay according to EFB. BB, button battery; EFB, esophageal foreign bodies.

There was a statically significant difference in the mean (SD) cost for higher-risk EFB [$6829 (11,347)] compared to lower-risk EFB [$2734 (10,451)] (*P* = .02). Button battery ingestions were more than twice as costly as all other higher-risk EFB and nearly five times as costly as all EFBs (Figure 4A). The mean (SD) LOS for button battery ingestions was 5.6 (6.8) days and all patients required at least two specialty consultations and repeat imaging either in the form of an X-ray, CT, or MRI. In comparison, the mean (SD) LOS overall for EFB ingestion was 1.1 (2.7) days (Figure 4B). For lower-risk foreign bodies, 33% were discharged directly from the PACU after removal, and 36% were admitted to the ward (Figure 1). Of those lower-risk foreign bodies who were admitted to the ward, 89% (145/163) were admitted for less than 24 hours. If these patients had been discharged directly from the PACU, this would have resulted in a cost savings of $2045 per patient, for a total of $296,525.

## Discussion

There are significant economic implications associated with the removal of impacted EFB in children. Beyond the costs associated with the surgical procedure of removing the EFB, there are associated costs such as hospital transfers, all of which increase exponentially when there are complications. These exert a substantial impact on healthcare resources. Canada spends more than 300 billion dollars annually on healthcare, 25% of which is attributable to hospital-related costs.^11^ With 30% of taxpayer revenues going to healthcare, it is important to understand where and how we can minimize this burden.^12^ Cost analysis studies like this are the first step in identifying actionable domains that can potentially be targeted.

The mean cost of EFB removal was $4265 for children transferred from a community hospital to a tertiary care hospital, but only $2300 when children were treated at the hospital they presented at. Increased costs were associated with increased time to the OR, driven primarily by repeat X-rays, delay in OHNS consultation, and higher-risk foreign bodies. Nearly 75% of all patients initially presented to a community hospital prior to being transferred to a tertiary care hospital. The province of Ontario is 1.1 million km^2^ and houses 3.5 million children below the age of 17.^13^ Variability in surgical expertise and capacity for pediatric care necessitates the transfer of children with EFB to specialized centers, of which there are only four in Ontario. Transfers from community hospitals are costly, as they often require a land or air ambulance, which can cost up to several thousand dollars per journey. Interestingly, only seven patients initially presented to a community hospital that did not have a pediatric medicine team and ward available. While the majority of community hospitals have available on-call general otolaryngologists, we were not able to account for the small minority of centers that did not as this is not publicly available. Given that the majority (76%) of EFB were not considered higher risk, it is conceivable that healthy children with lower-risk EFB could be treated at the presenting community hospital, thereby avoiding delays in care and increased costs associated with their transfer. Further investigation into reasons why patients were not treated at the initial community hospital are required. Building capacity through continuing medical education programs focused on EFB removal may allow lower-risk EFBs to be removed at their presenting hospitals, thereby avoiding transportation and removal delays.

Prolonged EFB impaction times have been associated with increased morbidity.^5,14^ In the present study, the average duration from assessment by an ED physician at a community hospital to contact with a tertiary care hospital was 3 hours, with a subsequent 2.4-hour delay from arrival at the tertiary care hospital to OHNS service contact. In this study, the latter corresponded to an average increase of $2000 compared to those who initially presented to a tertiary care hospital. Studies on the triage pathway and referral timeline for other common emergencies such as acute myocardial infarction or ischemic stroke have been shown to decrease delay in treatment time, improve patient outcomes, and lower costs.^15-17^ Within otolaryngology, a specific button battery protocol has shown significant decrease in button battery removal time.^18^ Early contact with the specialist team was a key factor in decreasing triage-to-treatment time, which can also be applicable to pediatric EFBs. Prioritizing discharge from the PACU after EFB removal is paramount, since children with lower-risk EFBs who were discharged from the PACU had an average $1735 decrease in overall cost compared to those admitted to the ward, resulting in a 77% decrease in cost. There was also no difference in complications or re-admission rates between these two groups. Similar cost savings have been reported in other studies advocating for outpatient surgery, such as knee arthroplasty and elective appendectomy, where outpatient surgery has been shown to decrease costs by 15% to 30% compared to inpatient surgery.^19-21^ Even for emergent general surgery, such as appendectomy, same day discharge has been shown to be safe and cost-effective in the Canadian system.^22^

Higher-risk EFBs were more likely to result in complications and prolonged hospital admissions in higher-care settings. The requirement for more intensive monitoring and interventions in cases involving higher-risk foreign bodies resulted in elevated costs ($6829 for higher-risk EFBs compared to $2734 for lower-risk EFBs per day). A single child with an esophageal button battery impaction can cost up to $180,000 for one admission, as was the case in one patient in this study. This may be explained by the 2015 North American Society for Pediatric Gastroenterology, Hepatology, and Nutrition guidelines on esophageal button battery management, which advocated for inpatient observation for complications and repeat MRIs during admission, resulting in higher cost burden and resource utilization with grossly unchanged patient outcomes.^23^ Other cost analyses for high-risk emergency surgeries have demonstrated significantly higher cost burdens when compared to their elective counterparts, especially in acute general surgery procedures, which can result in a 50% increase in cost.^24,25^ Upstream interventions such as educational programs in elementary schools or button battery safety petitions also need to be considered. Specifically for button batteries, Reese’s Law was passed in the United States in 2022 which mandated safety protocols for button battery packaging after a child’s death due to a button battery ingestion.^26^ In Canada, multiple provinces have established button battery protocols and guidelines aimed at minimizing morbidity and expediting care.^27,28^ Advocacy through awareness programs can also be implemented, similar to school-based sunscreen awareness programs which have shown an increase in reported sunscreen usage among youth and declining rates of sun-related skin cancers.^29-31^ Education surrounding EFB can be incorporated into elementary school curriculum or taught to parents at the level of primary care offices.

A limitation of this study is that we used a database from a tertiary pediatric care center, there may be a skew toward higher-risk EFBs. However, we found that 77% of the EFB transferred from the community were lower risk in nature. As we could not obtain data from the community hospitals, costs associated with transfer are likely underestimated. We also do not have the data for patients with EFBs who were managed at a community hospital, therefore a more comprehensive review of the province would also include those who underwent esophagoscopy at their community hospital. Given that this study was conducted in the province of Ontario, Canada with a healthcare model that may differ from other regions, this also may make our results difficult to generalize. Lastly, over the time period of this study, there may have been fluctuations in certain healthcare costs due to inflation, which we elected to not factor in for simplicity’s sake but nonetheless would be expected to have a role to play.

## Conclusion

EFB impose a significant economic burden on the healthcare system, largely driven by delays in care, transfers to tertiary care centers, and higher-risk EFBs. It is essential to identify areas for improved efficiency, such as upstream prevention campaigns in daycares and elementary schools, enhanced capability to manage low-risk EFBs at the presenting community hospital, early involvement of the OHNS team, and improved communication and transfer to the tertiary hospital for higher-risk EFB. We intend to use this targetable data as the foundation for change, to create more efficient, data-driven pathways within our healthcare system so that we can continue to improve care for children with EFB.

## Figures and Tables

**Figure A1.**
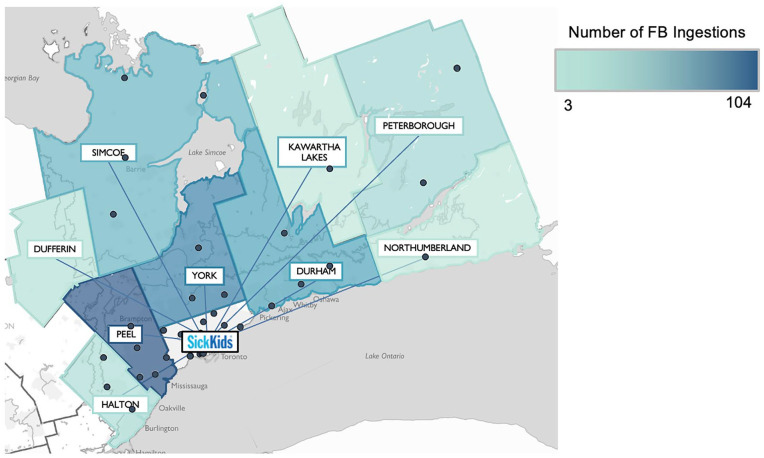
Geographic distribution of esophageal foreign body ingestion patterns across Ontario.
